# A breakdown in microglial metabolic reprogramming causes internalization dysfunction of α-synuclein in a mouse model of Parkinson’s disease

**DOI:** 10.1186/s12974-022-02484-0

**Published:** 2022-05-22

**Authors:** Jia Lu, Chenfei Wang, Xin Cheng, Ruizhi Wang, Xuehan Yan, Pengju He, Hongzhuan Chen, Zhihua Yu

**Affiliations:** 1 Department of Pharmacology and Chemical Biology, Shanghai Jiao Tong University School of Medicine, 280 South Chongqing Road, Shanghai, 200025 China; 2 Shanghai Jiao Tong University School of Medicine, Shanghai, 200025 China; 3 Shanghai University of Traditional Chinese Medicine, Shanghai, 201203 China

**Keywords:** TRPV1, α-Synuclein, Microglia, Autophagy, Capsaicin

## Abstract

**Background:**

The α-synuclein released by neurons activates microglia, which then engulfs α-synuclein for degradation via autophagy. Reactive microglia are a major pathological feature of Parkinson’s disease (PD), although the exact role of microglia in the pathogenesis of PD remains unclear. Transient receptor potential vanilloid type 1 (TRPV1) channels are nonselective cation channel protein that have been proposed as neuroprotective targets in neurodegenerative diseases.

**Methods:**

Using metabolic profiling, microglia energy metabolism was measured including oxidative phosphorylation and aerobic glycolysis. The mRFP-GFP-tagged LC3 reporter was introduced to characterize the role of TRPV1 in microglial autophagy. α-synuclein preformed fibril (PFF) TRPV1^*flox/flox*^; Cx3cr1^Cre^ mouse model of sporadic PD were employed to study the capacity of TRPV1 activation to attenuate neurodegeneration process.

**Results:**

We found that acute exposure to PFF caused microglial activation as a result of metabolic reprogramming from oxidative phosphorylation to aerobic glycolysis via the AKT–mTOR–HIF-1α pathway. Activated microglia eventually reached a state of chronic PFF-tolerance, ﻿accompanied by broad defects in energy metabolism. We showed that metabolic boosting by treatment with the TRPV1 agonist capsaicin rescued metabolic impairments in PFF-tolerant microglia and also defects in mitophagy caused by disruption of the AKT–mTOR–HIF-1α pathway. Capsaicin attenuated phosphorylation of α-synuclein in primary neurons by boosting phagocytosis in PFF-tolerant microglia in vitro. Finally, we found that behavioral deficits and loss of dopaminergic neurons were accelerated in the PFF TRPV1^*flox/flox*^; Cx3cr1^Cre^ mouse model of sporadic PD. We identified defects in energy metabolism, mitophagy and phagocytosis of PFF in microglia from the substantia nigra pars compacta of TRPV1^*flox/flox*^; Cx3cr1^Cre^ mice.

**Conclusion:**

The findings suggest that modulating microglial metabolism might be a new therapeutic strategy for PD.

**Supplementary Information:**

The online version contains supplementary material available at 10.1186/s12974-022-02484-0.

## Introduction

Parkinson’s disease (PD) is a multisystem neurodegenerative disease characterized by the appearance of Lewy bodies containing misfolded fibrillar α-synuclein and the selective loss of midbrain dopaminergic neurons in the substantia nigra pars compacta (SNpc), leading to postural instability, bradykinesia and tremor [1, 2]. Although little is known about the etiology of PD processes, autosomal dominant α-synuclein gene amplifications or mutations directly link α-synuclein dysfunction to PD causation [3, 4]. Moreover, dysfunctional α-synuclein pathology is present in both sporadic and familial PD patients and the distribution of α-synuclein correlates with the clinical symptoms of PD. Aggregated α-synuclein is the major component of Lewy bodies and is associated with neurodegenerative diseases, including PD and dementia with Lewy bodies [5]. The α-synuclein can be transmitted between neurons by a progressive “prion-like” mechanism as a response to stimulation or cellular stress [6, 7]. Neighboring glia cells can take up and clear extracellular α-synuclein to maintain α-synuclein homeostasis in the brain. The uptake and degradation processes of glia cells are thus key to regulating the spread and deposition of α-synuclein, and even to altering the pathological progression of PD.

Microglia, which are the prototypical scavenger cells in the brain, show the highest efficiency for ingesting and degrading extracellular α-synuclein in vitro [8]*.* It has been reported that α-synuclein released from neurons activates microglia, which then engulf and degrade α-synuclein via autophagy, both in vitro and in vivo [9]. However, the efficiency of the microglial autophagy–lysosome degradation system is reduced in PD, leading to accumulation of misfolded α-synuclein and degeneration of dopaminergic neurons [3, 10]. Increasing microglial phagocytosis and degradation of α-synuclein by autophagy may thus represent an important therapeutic target for PD.

Transient receptor potential vanilloid 1 (TRPV1) channels are nonselective ligand-gated cation channels that have been proposed as neuroprotective targets in neurodegenerative diseases such as Alzheimer’s disease and PD [11–13]. The TRPV1 agonist capsaicin was shown to prevent degeneration of dopaminergic neurons by inhibiting oxidative stress and neuroinflammation caused by activation of glia in the 1-methyl-4-phenyl-1,2,3,6-tetrahydropyridine model of PD [14]. Capsaicin was also shown to restore rotarod performance, as well as dopaminergic signaling, in the 1-methyl-4-phenyl-1,2,3,6-tetrahydropyridine model of PD [15]. It was further demonstrated that the neuroprotective effects of TRPV1 activation using capsaicin were mediated through endogenous ciliary neurotrophic factor and its receptors [12].

Here, we identified defects in energy metabolism, mitophagy and phagocytosis in microglia with chronic tolerance to α-synuclein preformed fibril (PFF). Metabolic boosting with capsaicin reversed metabolic impairments and mitophagy defection in PFF-tolerant microglia by modulating the AKT–mTOR–HIF-1α pathway. We showed that behavioral deficits and loss of dopaminergic neurons were accelerated in the α-synuclein PFF TRPV1^*flox/flox*^; Cx3cr1^Cre^ mouse model of sporadic PD. In light of its beneficial effects, TRPV1 activation should be evaluated for the treatment of PD and related neurodegenerative disorders characterized by activation of microglia.

## Materials and methods

### Mice

TRPV1^*flox/flox*^ mice were obtained from the Shanghai Model Organisms Center, Inc. (Shanghai, China). Cx3cr1^Cre^ transgenic mice (no. 021160) were purchased from the Jackson Laboratory (Bar Harbor, ME, USA). TRPV1^*flox/flox*^ mice were bred with Cx3cr1^Cre^ mice to generate TRPV1^*flox/flox*^; Cx3cr1^Cre^ mice. Eight-week-old male and female TRPV1^*flox/flox*^; Cx3cr1^Cre^ mice were injected intraperitoneally with tamoxifen (sc-208414, Santa Cruz Biotechnology, Inc., Santa Cruz, CA, USA) at a dose of 75 mg/kg/day for 5 consecutive days. The mice were housed at room temperature (22 ± 1 °C) under a 12-h light/dark cycle. The protocols for all animal experiments were approved by the Animal Experimentation Ethics Committee of Shanghai Jiao Tong University School of Medicine (Shanghai, China).

### Preparation of α-synuclein PFF

Recombinant human α-synuclein proteins were purchased from ATgen (SNA2001L, ATgen, Seongnam, South Korea). α-Synuclein monomers were dissolved in phosphate-buffered saline (PBS) at a concentration of 5 mg/ml, and incubated for 7 days (1000 rpm at 37℃) [16]. Aggregates should be shipped and stored at − 80℃. Samples were diluted to the desired concentration and sonicated for 30 s at 10% amplitude immediately prior to use [17]; solutions were mixed between injections and used within 4 h. Successful generation of α-synuclein PFF was validated by transmission electron microscopy (TEM) and the ability to generate p-α-syn (ser129) pathology.

### Transmission electron microscopy

The morphology of α-synuclein monomers, PFF and sonicated PFF was monitored by TEM. Specimens were prepared by depositing on 75 mesh copper grids coated with Formvar and carbon. The grids were washed after 5 min and negatively stained with 2% uranyl acetate for 30 s. Excess liquid was wicked away using a filter paper and the grids were dried in the air at room temperature for 20 min. Images was captured using an H-7650 transmission electron microscope (Hitachi, Tokyo, Japan).

### Stereotaxic injection of α-synuclein PFF

Littermates of TRPV1^*flox/flox*^ and TRPV1^*flox/flox*^; Cx3cr1^Cre^ were randomly allocated to experimental groups at the age of 8–12 weeks. For PFF delivery, mice were anaesthetized and placed in a stereotaxic frame (RWD Life Science Co., Ltd., Shenzhen, China). A solution of PFF in PBS (2 μL, 2.5 μg/μL) or an equal volume of PBS was injected unilaterally into the striatum (right hemisphere) at a rate of 0.2 µL/min using an injection pump. In keeping with a previous report [17], the injection coordinates were: mediolateral, 2.0 mm from bregma; anteroposterior, 0.2 mm; dorsoventral, 2.6 mm. The needle was left in the striatum for 10 min to allow the injectant to diffuse before it was slowly removed and the wound sutured.

### Behavioral test

In order to evaluate the impact of PFF on motor coordination, mice were assessed by rotarod test and open field test 4 months after stereotactic injection of PBS and PFF into striatum. All the in vivo experiments were double-blind. The open field test and rotarod test was carried out as described previously [18].

#### Open field test

The animals were acclimated to the environment for at least 1 day prior to initiating experiments. Then, the test mice were placed in the center of the open field room (40 cm × 40 cm × 40 cm) and was permitted to move freely for 5 min. Their locomotor activity was recorded by a video camera and analyzed via a tracking system (Noldus Ethovision, Wageningen, Netherlands). At the end of each trail, the chamber was cleaned with 75% ethanol solution to remove urine or odor.

#### Rotarod test

After the open field test, mice were acclimated to the rotarod apparatus (IITC Life Science, Woodland Hills, CA, USA) for 150 s on two consecutive days at a low rotation speed of 8 rpm and 12 rpm. While on the seven consecutive testing days, the mice were placed on the rotating rods at 8, 12, 16, 20, 24, 28 rpm. The maximum time for each trail was 150 s. The time spent on the rod was recorded and the area under the curve was calculated to evaluate the motor coordination. Mice were rested for 30 min between trails to avoid influence of exhaustion and stress.

#### RNA sequencing analysis

In this study, total RNA was extracted from collected hemibrain tissues. The mRNA with poly A structure were enriched by Oligo (dT) magnetic beads in the total RNA, and then interrupted about 300 bp fragment by ion interruption. The first strand of cDNA was synthesized by using reverse transcriptase as template, 6 base random primers, and the second strand cDNA was synthesized using the first-strand cDNA as a template. The library fragments were enriched by PCR amplification after cDNA libraries construction, and the libraries were selected according to the fragment size (450 bp). The cDNA libraries were validated using the Agilent 2100 Bioanalyzer (Agilent Technologies, Palo Alto, CA, USA). Libraries were sequenced on the Illumina sequencing platform by Next-Generation Sequencing. The FASTQ raw data were filtered to remove the reads with adapters and low-quality sequences, and mapped to the mouse reference genome using HISAT2 (http://ccb.jhu.edu/software/hisat2/index.shtml). Gene expression counts were calculated according to comparison results. R pheatmap package (https://cran.rproject.org/web/packages/pheatmap/index.html) was used to perform bi-directional clustering analysis of all different genes of samples. Then, we used DESeq. (1.30.0) to identify the differential gene expression between samples according to screening conditions: absolute value of log2 (fold change) larger than 1 and significance *p* value < 0.05. Volcano plots were generated using R ggplots2 package. Gene enrichment analyses were performed using Kyoto Encyclopedia of Genes and Genomes (KEGG) (http://www.kegg.jp/) with default parameters to determine the biological functions of the differential expression genes.

### Weighted gene co-expression network analyses

Weighted gene co-expression network analyses (WGCNA) were performed to identify the effects of microglial TRPV1 on PD-like pathology induced by PFF using the R WGCNA package [19] (WGCNA version 1.66, https://cran.r-project.org/src/contrib/Archive/WGCNA/). Clustering analyses were performed on the gene expression. Afterward, the TOMSimilarity module was used to calculate the gene–gene co-expression similarity coefficient, which was converted to connections among the genes by the pickSoftThreshold function, and to realize the functional connection of genes. Next, a weighted co-expression network model was established to divide thousands of genes into several modules. The correlation coefficient and *p* value were calculated and screened modules related to the biological characteristics. Genes in several modules were significantly enriched into pathways of both Gene Ontology (GO) and KEGG using Metascape (https://metascape.org).

### Immunohistochemistry

The mice were anaesthetized and transcardially perfused with PBS. Coronal sections of the SNpc (10 μm) were incubated with rabbit anti-p-α-syn (Ser129) (1:500, 23706S; Cell Signaling Technology, Beverly, MA, USA) at 4 ℃ overnight. The slices were then incubated with biotinylated anti-rabbit IgG, treated with avidin–biotin peroxidase complex, and visualized using 0.05% DAB + 0.03% H_2_O_2_. Images were captured using a DM6 B microscope (Leica Microsystems, Wetzlar, Germany), and p-α-syn (Ser129) positive cells in the brain region were counted by ImageJ software (NIH, Bethesda, USA).

### Immunofluorescence and quantitative analysis

Brain tissues of the SNpc were permeabilized with 0.3% Triton X-100, and blocked with 10% goat serum in 0.01 M PBS for 1 h at room temperature. Brain sections were incubated with the following primary antibodies at 4℃ overnight: rabbit anti-TH (1:500, ab112, Abcam, Cambridge, UK), mouse anti-Iba-1 (1:500, GB12105, Servicebio Technology Co., Ltd., Wuhan, China), rabbit anti-p-α-syn (Ser129) (1:500, 23706S; Cell Signaling Technology). The slices were next incubated with Alexa Fluor 647 goat anti-mouse (1:1000, A32728; ThermoFisher Scientific, Waltham, MA, USA) and Alexa Fluor 568 goat anti-rabbit (1:500, A21428, ThermoFisher Scientific), and then stained with 4′,6-diamidino-2-phenylindole (DAPI). Fluorescent images were captured using a TCS SP8 confocal laser scanning microscope (Leica Microsystems). Three-dimensional reconstructions of microglia were performed using Imaris software (BitPlane Scientific Software, Zürich, Switzerland), and subsequent analyses were conducted using Fiji software (NIH, Bethesda, USA), as previously described [20]. Analysis of microglial morphology was conducted using the Skeletonize plug-in for Fiji.

### Culture and stimulation of primary microglia

Primary cultures of microglia were prepared from the cerebral cortices and hippocampi of newborn (P0–P2) C57BL/6 mice. The brain tissues were dissociated into single cells by treatment with 0.25% trypsin for 10 min at 37 ℃, and the cells were then cultured in high glucose Dulbecco’s Modified Eagle’s medium (DMEM) (SH30022.01B; Hyclone, Beijing, China), supplemented with 10% fetal bovine serum (S711-001S; Shuangru Biotechnology Co., Ltd., Shanghai, China), 1% penicillin/streptomycin (C0222; Beyotime Institute of Biotechnology, Shanghai, China) and 2 mM L-glutamine (C0212; Beyotime Institute of Biotechnology) at 37℃ in an incubator with 5% CO_2_. On day 14, the microglia were harvested from the mixed glial culture by shaking at 200 rpm for 4 h at 37 ℃. Primary microglia were plated and incubated for 3–4 days before using.

Primary microglia were stimulated with 100 ng/ml lipopolysaccharide (LPS) (055: B5; Sigma-Aldrich, St. Louis, MO, USA), or 1 μg/ml PFF for 24 h. In some experiments, microglia were pretreated with 30 nM rapamycin (TargetMol, Shanghai, China) to block the mTOR pathway or 10 μM capsaicin (TargetMol, Shanghai, China) to activate the TRPV1 channel before addition of PFF. Experiments using the tolerant model were used to mimic chronic disease. Primary microglia were incubated with 1 μg/mL PFF for 24 h, the medium was removed and replaced with culture medium and the cells were incubated for a further 3–5 days. The cells were then restimulated with 1 μg/mL PFF for 24 h. For treatment experiments, tolerant microglia were pretreated with 10 μM capsaicin for 30 min and then 1 μg/mL PFF was added. Primary microglia-conditioned medium (MCM) from cells pretreated with 10 μM capsaicin and then treated with 1 μg/ml PFF was collected and stored at − 80 ℃.

### Primary culture of cortical neurons

Primary cortical neurons were isolated from postnatal Day 0 C57BL/6 J mouse pups as described previously [18]. Briefly, mouse cortices were dissected in ice-cold DMEM and then dissociated into single-cell suspensions by treatment with papain (A501612-0025; Sangon Biotech, Shanghai, China). After dissociation, the neurons were seeded on poly-L-lysine-coated dishes (E607015; Sangon Biotech). After 6–8 h, plating medium (DMEM supplemented with 10% horse serum (E510006-0100; Sangon Biotech), 1% glutamax (35,050,061; Gibco, Grand Island, NY, USA) and 1% penicillin/streptomycin) was replaced with Neurobasal medium (T710KJ; BasalMedia Technologies Co., Ltd., Shanghai, China) containing 2% B27 supplement (S440J7; BasalMedia Technologies) and 1% glutamax. Neurons were grown in vitro for 7 days, with the medium changed every 3 days. Primary cortical neurons were pretreated with 5 μg/ml PFF for 5 days and then treated with 50% PFF MCM for 24 h.

### Quantitative real-time PCR

Total RNA was isolated from microglia using TRIzol reagent (R0016; Beyotime Institute of Biotechnology). The first-strand of cDNA was synthesized by reverse transcription using a cDNA synthesis kit (6210A; Takara Ltd., Otsu, Japan) after examining total RNA using a BioDrop spectrophotometer (Biochrom Ltd, Cambridge, UK). Quantitative real-time PCR (qPCR) with 10 ng diluted cDNAs was performed on a LightCycler 480II (Roche Applied Science, Mannheim, Germany), using BeyoFast™ SYBR Green qPCR Mix (D7265, Beyotime Institute of Biotechnology). The qPCR was carried out with a hold step at 95 ℃ for 2 min; 40 cycles of denaturation at 95 ℃ for 15 s, annealing at 60 ℃ for 20 s and extension at 72 ℃ for 30 s. Melting curve analysis was performed from 60 to 95 ℃. Expression levels of target genes were normalized to GAPDH and calculated using the 2^−ΔΔCt^ method. The primer sequences were as follows: for IL-1β, 5'-TCCAGGATGAGGACATGAGCAC-3' (forward) and 5'-GAACGTCACACACCAGCAGGTTA-3' (reverse); for TNF-α, 5'-CAGGAGGGAGAACAGAAACTCCA-3' (forward) and 5'-CCTGGTTGGCTGCTTGCTT-3' (reverse); for GAPDH, 5'-TTGATGGCAACAATCTCCAC-3' (forward) and 5'-CGTCCCGTAGACAAAATGGT-3' (reverse).

### Western blotting

Primary microglia and the SNpc region of mouse brain were homogenized and prepared in radioimmunoprecipitation lysis buffer (P0013B, Beyotime Institute of Biotechnology) on ice containing phenylmethanesulphonyl fluoride. Lysates containing equal concentration of 50 μg proteins were separated by 8%, 12% or 15% (*w/v*) sodium dodecyl sulfate–polyacrylamide gel electrophoresis and transferred to a polyvinylidene difluoride membrane, which was then blocked in 5% nonfat milk at room temperature for 1 h. The membranes were incubated at 4℃ overnight with indicated antibodies as following: TH (1:1000, ab112, Abcam), Iba-1 (1:1000, 019-19741, Wako Pure Chemical Industries, Ltd., Osaka, Japan), TRPV1 (1:200, ACC-030, Alomone labs, Jerusalem, Israel), p-α-syn (Ser129) (1:1000, 23706S, Cell Signal Technology), α-synuclein (1:1000, 4179T, Cell Signal Technology), GFAP (GA5) (1:1000, 3670T, Cell Signal Technology), Phospho-mTOR (Ser2448) (1:1000, 5536T, Cell Signal Technology), mTOR (1:1000, 2983T, Cell Signal Technology), Phospho-Akt (Thr308) (1:1000, 2965P, Cell Signal Technology), Akt (1:1000, 4691P, Cell Signal Technology), Phospho-AMPKα (Thr172) (1:1000, 2535T, Cell Signal Technology), LC3B (1:1000, 2775, Cell Signal Technology), SQSTM1 (1:1000, 5114S, Cell Signal Technology), Atg3 (1:1000, 3415T, Cell Signal Technology), β-actin (1:1000, 3700S, Cell Signal Technology), HIF1-α (1:500, AH339-1, Beyotime Institute of Biotechnology), Pink1 (1:1000, AF7755, Beyotime Institute of Biotechnology), Parkin (1:1000, AF7680, Beyotime Institute of Biotechnology), p-TBK1/NAK (Ser 172) (1:500, AF5959, Beyotime Institute of Biotechnology). Afterward, the membranes were incubated with the peroxidase-conjugated anti-mouse (1:1000, A0216, Beyotime Institute of Biotechnology) and anti-rabbit (1:1000, A0208, Beyotime Institute of Biotechnology) IgG at room temperature for 1 h. Immunoreactive proteins were visualized using an enhanced chemiluminescent substrate (36222ES76, Yeasen, Shanghai, China) and the Image Studio Lite Ver 5.2 software (LI-COR Biosciences, Lincoln, NE, USA).

### Metabolic assay

Bioenergetic analysis of microglia was performed using a Seahorse XFe96 analyzer (Seahorse Bioscience, Billerica, MA, USA) on XF96 cell culture microplates (102416-100; Seahorse Bioscience). Before the assay, primary microglia were seeded at a density of 10,000 cells/well and cultured overnight. After drug treatment, the medium was replaced with Seahorse XF Base medium (102353-100; Seahorse Bioscience) containing 25 mM glucose (G7528, Sigma-Aldrich), 200 mM glutamine (2503164; Gibco) and 1 mM pyruvate (11360070; Gibco), and the microglia were incubated in a 37℃ incubator without CO_2_. Mitochondrial stress was induced by sequential injection of 5 μM oligomycin, 10 μM carbonyl cyanide p-(trifluoromethoxy) phenyl-hydrazone and 10 μM rotenone and antimycin A (103015-100; Seahorse Bioscience). Glycolysis stress was detected by sequential injection of 10 mM glucose, 0.5 μM oligomycin and 50 mM 2-deoxy-glucose (103020-100; Seahorse Bioscience). Raw data were analyzed and exported using Wave 2.6.0 version (Agilent Technologies, Inc., Santa Clara, CA, USA).

### Measurement of reactive oxygen species

Primary microglia were incubated with 1 μg/mL PFF, with or without 10 μM capsaicin, for 24 h. Production of reactive oxygen species (ROS) was detected by 2′,7′-dichlorofluorescein diacetate (10 μM) staining, using a fluorescence microplate reader (ThermoFisher Scientific) with detection at 450 nm.

### Measurement of mitochondrial membrane potential

Microglia were seeded and cultured overnight in glass bottom dishes at a density of 1 × 10^5^ cells/well. Mitochondrial membrane potential was measured using a MitoProbe™ JC-1 Assay Kit (C2005; Beyotime Institute of Biotechnology). Microglia were incubated with a 10 μM solution of JC-1 dye for 20 min at 37 °C (shielded from light) and washed with PBS prior to assessment using a TCS SP8 confocal laser scanning microscope. In normal mitochondria, JC-1 forms aggregates, which emit a red fluorescence (561 nm). Following decline or loss of mitochondrial membrane potential, aggregated JC-1 was released into the cytoplasm as the monomeric form, which emits a green fluorescence (488 nm). Mitochondrial membrane potential was calculated as the red/green ratio using ImageJ software.

### Cellular uptake of fluorescent beads

Phagocytic ability was measured using a fluorescent beads uptake assay, as previously described [21]. Fluorescent latex beads (1 μM, L2778; Sigma-Aldrich) were pretreated with 50% fetal bovine serum, centrifuged at 1,000 rpm for 2 min, and then diluted with serum-free DMEM. The preprocessed beads were loaded onto microglia and incubated at 37 °C for 4 h. Residual beads were washed off the cells with PBS at the end of incubation period and the microglia were then fixed and stained with DAPI. Microglial phagocytic uptake of the fluorescent latex beads was determined using a TCS SP8 confocal laser scanning microscope with ImageJ software.

### Measurement of autophagy flux

Tandem fluorescent mRFP-GFP-LC3 plasmids were used to label and track LC3 and measure the autophagic flux. In brief, cultured primary microglia were transfected with mRFP-GFP-LC3 plasmids (Addgene, Watertown, MA, USA), according to the manufacturer’s protocol, and incubated for 48 h. The microglia were then treated with PFF or capsaicin for 24 h before fixing and staining with DAPI. The locations of mRFP and GFP were tracked using a TCS SP8 confocal laser scanning microscope. The autophagic flux was evaluated by counting the puncta of different colors with ImageJ software.

### Measurement of mitochondrial autophagy

After PFF or capsaicin stimulation, cells were incubated in the dark with 200 nM MitoTracker Red CMXRos (C1049B; Beyotime Institute of Biotechnology) at 37 °C for 20 min. The microglia were blocked with 10% goat serum in 0.01 M PBS, and incubated with mouse anti-LC3B (1:400, 83506S; Cell Signaling Technology) or rabbit anti-Parkin (1:200, AF7680; Beyotime Institute of Biotechnology) at 4 °C overnight. The cells were stained with Alexa Fluor 647 goat anti-mouse (1:1000, A32728; ThermoFisher Scientific) or Alexa Fluor 647 goat anti-rabbit (1:500, A21245; ThermoFisher Scientific), and then stained with DAPI. Colocalization of fluorescence was captured using a TCS SP8 confocal laser scanning microscope and analyzed by ImageJ software.

### Immunofluorescence of primary cortical neurons

Primary cortical neurons were treated with 5 μg/mL PFF for 5 days and then incubated with 50% MCM for 24 h. The cells were blocked with 10% goat serum, and incubated with mouse anti-MAP2 (1:500, ab254143; Abcam) and rabbit anti-p-α-syn (Ser129) (1:500, 23706S; Cell Signaling Technology) at 4 °C overnight. The cells were stained with Alexa Fluor 647 goat anti-mouse (1:1,000, A32728; ThermoFisher Scientific) and Alexa Fluor 568 goat anti-rabbit (1:500, A21428; ThermoFisher Scientific), and then stained with DAPI. Fluorescent images were captured using a TCS SP8 confocal laser scanning microscope and analyzed by ImageJ software.

### Statistical analysis

All data are expressed as mean ± SD. Statistical analyses were performed using GraphPad Prism 7.0 (GraphPad Software, Inc., La Jolla, CA, USA). The Kolmogorov–Smirnov normality test was performed to test if the values fit a Gaussian distribution. The Student’s *t*-test was used to compare two independent groups. One-way analysis of variance (ANOVA) and Tukey’s multiple comparisons test were used to compare three or more independent groups. Two-way ANOVA with a Bonferroni post-test was used to compare multiple factors. Statistical significance was set at *p* < 0.05. Sample sizes were estimated from pilot experiments.

## Results

### Acute microglial inflammation induced by PFF is accompanied by metabolic reprogramming from oxidative phosphorylation to aerobic glycolysis

**Fig. 1:**
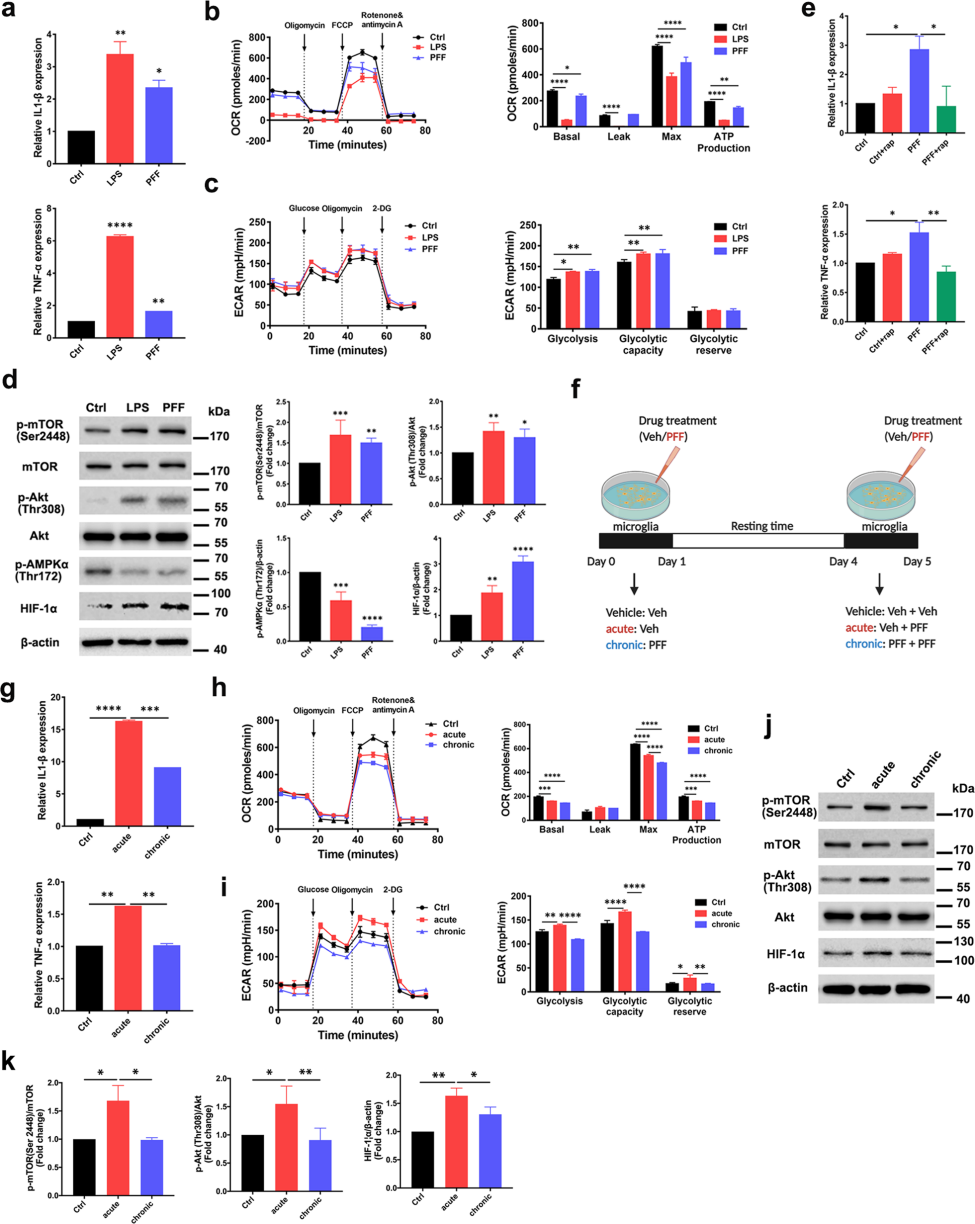
Chronic treatment with PFF causes innate immune tolerance and metabolic defects in microglia via mTOR pathway. **a** Relative mRNA expression of IL-1β and TNF-α in microglia treated with control, LPS, or PFF for 24 h (*n* = 3 per group). **b** Oxidative phosphorylation determined by measuring OCR and **c** glycolysis determined by measuring ECAR in microglia treated with vehicle, LPS or PFF for 24 h (*n* = 3 per group). **d** Protein expression levels of mTOR, p-mTOR, AKT, p-AKT, p-AMPK, HIF-1a and β-actin in microglia treated with LPS or α-syn PFF (n = 5, biological replicates). **e** Relative mRNA expression of IL-1β and TNF-α in microglia treated with PFF only or with PFF and rapamycin for 24 h (n = 3 per group). **f** Schematic illustration of in vitro experiments to investigate effects of acute or chronic treatment with PFF. **g** Relative mRNA expression of IL-1β and TNF-α in microglia after treatment with control, acute PFF or chronic PFF (*n* = 3 per group). **h** Oxidative phosphorylation determined by measuring OCR and **i** glycolysis determined by measuring ECAR in microglia after treatment with control, acute PFF or chronic PFF (*n* = 3 per group). **j**, **k** Immunoblot analysis of mTOR pathway components in microglia after treatment with control, acute PFF or chronic PFF (*n* = 3–5 per group). One-way ANOVA with Tukey’s multiple comparisons test was used for statistical analysis. Error bars represent mean ± SD. *p < 0.05, **p < 0.01, ***p < 0.001, ****p < 0.0001

To investigate the response of microglia to PFF, microglia were treated with PFF or *E. coli*-derived LPS for 24 h. First, mRNA expression of the proinflammatory cytokines IL-1β and TNF-α was investigated following stimulation with PFF and LPS. Both PFF and LPS substantially increased IL-1β and TNF-α mRNA expression (Fig. 1a). These data suggested that PFF stimulated an acute inflammatory response in microglia, which was associated with an enhancement of immune responses, demonstrating that PFF was recognized by microglia as a danger signal.

Given that the status of immune cells is reflected by their cellular metabolism, the metabolic dynamics of microglia were monitored by mitochondrial function and glycolysis. The oxygen consumption rate (OCR) was used to measure mitochondrial function after treatment with the ATP synthase inhibitor oligomycin, the carbonyl cyanide p-(trifluoromethoxy) phenylhydrazone, or rotenone and electron-transport chain inhibitor antimycin A, respectively. The OCR experiments revealed that PFF significantly downregulated both the basal and maximal respiratory capacity of mitochondria, as well as downregulating ATP production (Fig. 1b). The extracellular acidification rate (ECAR) increased after exposure to PFF or LPS, indicating a metabolic shift toward glycolysis (Fig. 1c). These data indicated that the metabolic status shifted from oxidative phosphorylation to glycolysis on PFF-induced microglial inflammatory activation.

The mTOR–AKT pathway is involved in regulating glucose metabolism as part of the mechanism for sensing cellular energy status. AMP-activated protein kinase (AMPK), a sensor for AMP and ADP, which represent metabolic exhaustion, inhibits mTOR phosphorylation. mTOR phosphorylation upregulates expression of HIF-1α, which is the transcriptional regulator of glycolysis [22]. The AKT–mTOR–HIF-1α and AMPK–mTOR–HIF-1α pathways were assessed following treatment with PFF to determine whether the mTOR energetic pathway was involved in PFF-induced metabolic reprogramming. As shown in Fig. 1d, treatment with PFF increased expression of p-AKT, p-mTOR and HIF-1α, and reduced expression of p-AMPK. Rapamycin, an allosteric inhibitor of mTOR, reduced PFF-induced mRNA expression of IL-1β and TNF-α (Fig. 1e). Collectively, these results indicated that microglial inflammation and altered metabolic phenotype following exposure to PFF was dependent on the mTOR/HIF-1α pathway.

### PFF induces microglial innate immune tolerance and metabolic defects

Because PD is a chronic and incurable neurodegenerative disease [23], microglia are activated at an early state and are, relatively, in a resting state during the interval of nigral degeneration in the SNpc [24]. Peripheral immune cells became activated within 1 day and then chronic immune tolerance developed during the next 3–5 days of exposure to danger signals [25]. This situation was mimicked by exposing microglia to acute PFF stimulation for 24 h, followed by further culture for 3–5 days without PFF stimulus. Accordingly, microglia were stimulated with PFF for 24 h, followed by further culture for 3–5 days after washing out the PFF (acute stimulation, Fig. 1f). A second group of microglia was then restimulated with PFF for 24 h (chronic stimulation). The mRNA levels of the pro-inflammatory cytokines IL-1β and TNF-α were lower in the chronically stimulated cells than in the acutely stimulated cells (Fig. 1g). We assessed glycolysis and oxidative phosphorylation metabolism, as well as mTOR–AKT pathway activity, to investigate the role of cellular metabolism in the impairment of immune function in microglia with tolerance to PFF induced by chronic exposure. Both ATP production and basal and maximal respiratory capacity of mitochondria were significantly reduced in microglia with chronic PFF-induced tolerance, compared with those with acute PFF-induced inflammation (Fig. 1h). Microglia with chronic PFF-induced tolerance showed significantly reduced glycolysis, glycolytic capacity and glycolytic reserve compared with microglia with acute PFF-induced inflammation (Fig. 1i). Proteins in the AKT–mTOR–HIF-1a pathway were also downregulated in microglia with chronic PFF-induced tolerance compared with those with acute PFF-induced inflammation (Fig. 1j, k). Western blotting analysis showed a downregulation in the level of p-AMPKα (Thr172) in microglia with chronic PFF-induced tolerance compared with those with acute PFF-induced inflammation (Additional file 1: Fig. S1a, b).

Taken together, these data indicated that comprehensive cellular metabolic defects, including changes in oxidative phosphorylation and aerobic glycolysis, were present in chronic PFF-induced tolerance, leading to immune dysfunction of microglia.

### Capsaicin rescues impaired cellular metabolism, mTOR signaling, and immune functions of PFF-tolerant microglia

**Fig. 2:**
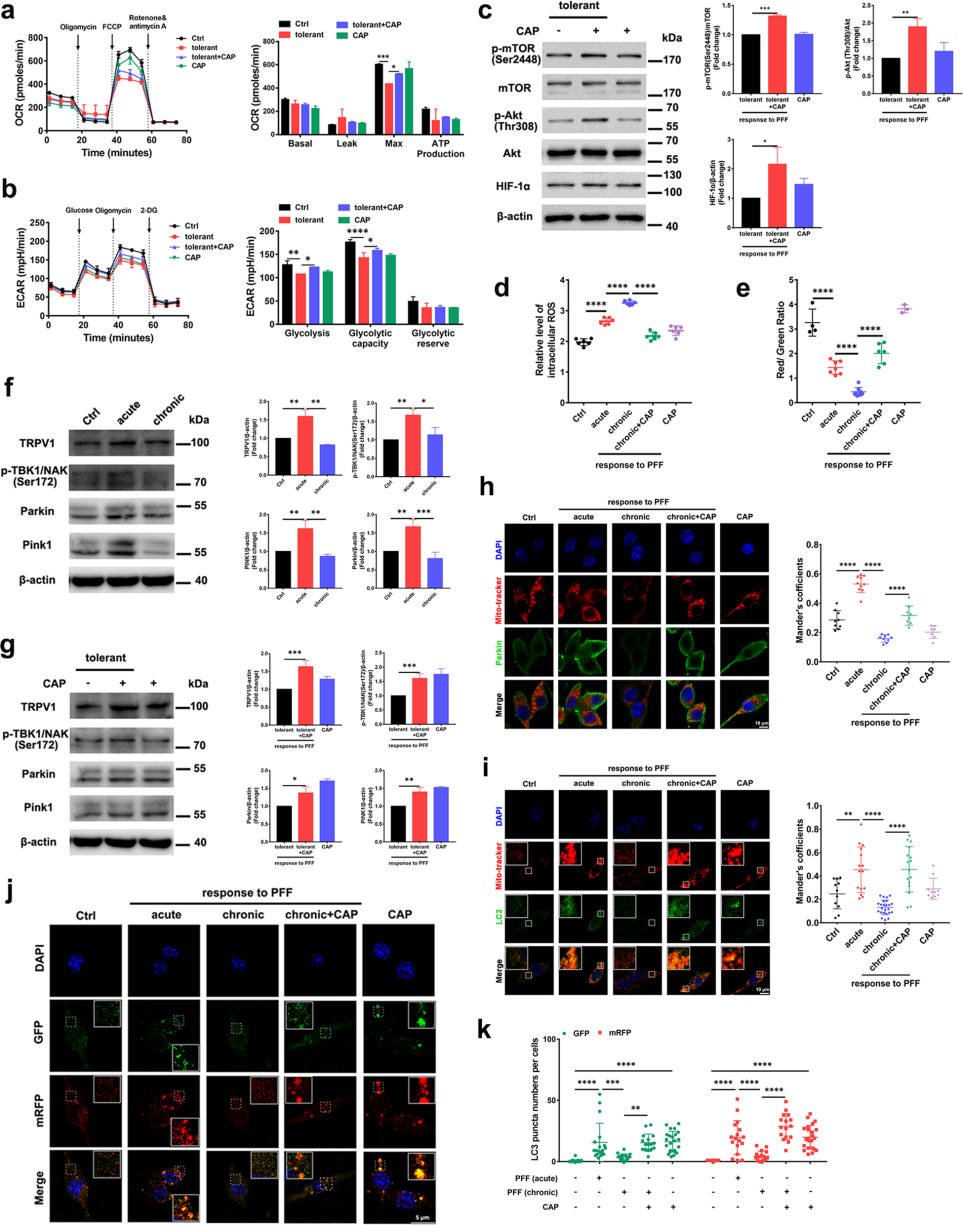
Capsaicin rescues impaired cellular metabolism and mitophagy via mTOR pathway in PFF-tolerant microglia. **a** Oxidative phosphorylation determined by measuring OCR and **b** glycolysis determined by measuring ECAR in tolerant PFF-treated microglia after exposure to vehicle or capsaicin (*n* = 3 per group). **c** Immunoblot analysis of mTOR pathway components in tolerant PFF-treated microglia after exposure to vehicle or capsaicin (*n* = 3 per group). **d** Quantification of mitochondrial membrane depolarization monitored by JC-1 MitoProbe (*n* = 6, biological replicates). **e** Intracellular ROS levels in microglia (*n* = 3–7, biological replicates). **f**, **g** Immunoblots and quantification of TRPV1, p-TBK1/NAK (Ser 172), Parkin, PINK1 and β-actin in PFF-tolerant cells after incubation with capsaicin (*n* = 3, biological replicates). **h**, **i** Representative fluorescent images and quantification of percentage colocalization of Mito-tracker (red) with Parkin (green) **h** or LC3 (green) **i** in PFF-tolerant microglia after incubation with capsaicin (*n* = 3, biological replicates). Scale bar, 10 μm. **j** Representative fluorescent images and **k** quantification of LC3 puncta examined using mRFP-GFP-LC3 plasmid transfection for 48 h in microglia (*n* = 3, biological replicates). Scale bar, 5 μm. One-way ANOVA with Tukey’s multiple comparisons test was used for statistical analysis. Error bars represent mean ± SD. *p < 0.05, **p < 0.01, ***p < 0.001, ****p < 0.0001

In line with the concept that microglia in PD show immune dysfunction because of broad metabolic defects, we hypothesized that repair of metabolic function might rescue microglial function in PD. Capsaicin was used to test whether metabolic dysfunction of PFF-tolerant microglia could be attenuated by metabolic boosting. Compared with untreated cells, oxidative phosphorylation (Fig. 2a) and glycolysis (Fig. 2b) were increased after pretreatment of PFF-tolerant microglia with 10 μΜ capsaicin, leading to reduced metabolic defects in the AKT–mTOR–HIF-1a pathway, including upregulation of phosphorylated AKT and mTOR, as well as increased HIF-1a levels (Fig. 2c). Intracellular ROS were measured using the molecular probe 2′,7′-dichlorofluorescein diacetate to determine the role of capsaicin in PFF-induced mitochondrial dysfunction. As shown in Fig. 2d, capsaicin reduced ROS upregulation in stimulated PFF-tolerant microglia (Fig. 2d). The mitochondria function indicator JC-1 was used to measure mitochondrial membrane potential. Capsaicin attenuated mitochondrial depolarization of stimulated PFF-tolerant microglia (Fig. 2e).

Previous studies revealed that mitophagy, a process of selective autophagy to remove dysfunctional or damaged mitochondria, played a vital role in maintaining complete mitochondrial network function, oxidative balance and cell survival [26, 27]. Accordingly, we investigated the possible role of mitophagy in regulation of mitochondrial function in PFF-tolerant microglia. Expression levels of TRPV1 and mitophagy markers p-TBK1/NAK (Ser172), Pink1 and Parkin were significantly downregulated in PFF-tolerant microglia compared with in cells treated acutely with PFF (Fig. 2f). As shown in Fig. 2g, capsaicin restored mitophagy in PFF-tolerant cells (Fig. 2g). The mitochondrial marker Mito-tracker Red was used to rigorously examine the process of mitophagy. The overlap coefficients between Parkin and Mito-tracker (Fig. 2h), and between LC3 and Mito-tracker (Fig. 2i), were significantly reduced in PFF-tolerant cells, indicating hindered initiation of mitophagy. Treatment with 10 μM capsaicin significantly increased the overlap coefficient between Parkin and Mito-tracker (Fig. 2h) and between LC3 and Mito-tracker (Fig. 2i). We also introduced the mRFP-GFP-tagged LC3 reporter into microglia to monitor the effect of capsaicin on autophagic flux. The fluorescence of green fluorescent protein (GFP), which is sensitive to the acidic pH inside the lysosome, was observed prior to fusion of autophagosomes with lysosomes, whereas only red (mRFP) fluorescence was present in post-fusion autolysosomes, which are resistant to acidic pH [28]. The number of red dots was significantly lower in PFF-tolerant microglia than in acutely stimulated microglia, indicating downregulation of autophagic flux. The number of red dots was significantly increased by pretreatment of PFF-tolerant microglia with 10 µM capsaicin, illustrating upregulation of autophagy flux (Fig. 2j, k).

These data demonstrated that treatment of PFF-tolerant microglia with capsaicin boosted the impaired AKT–mTOR–HIF-1a pathway and energetic metabolism, including oxidative phosphorylation and glycolytic metabolism, as well as restoring PFF-induced mitophagy function.

### Capsaicin attenuates phosphorylation of α-synuclein in primary neurons by boosting the immune function of tolerant microglia in vitro

**Fig. 3:**
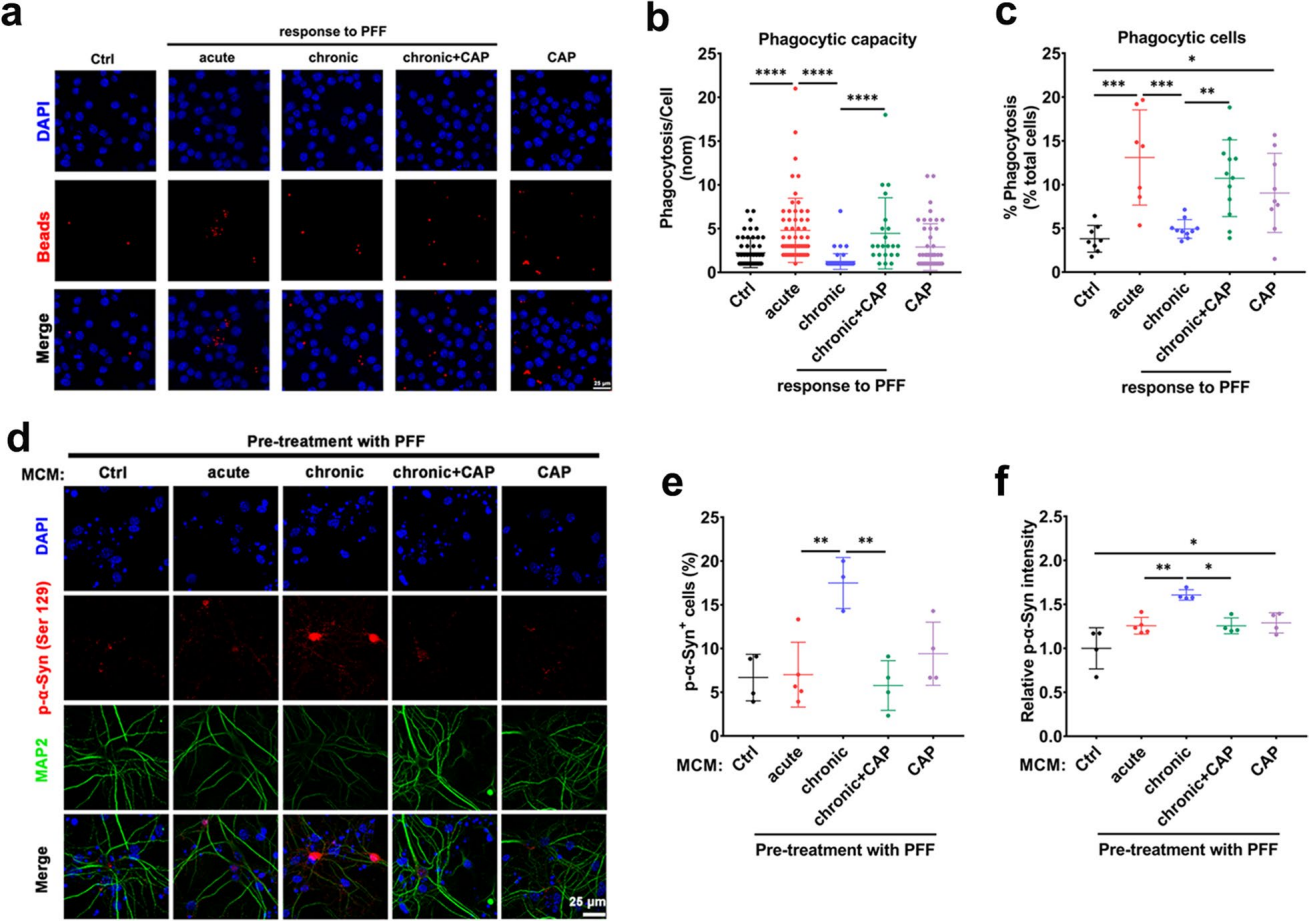
Capsaicin attenuates phosphorylation of α-synuclein in primary neuron by boosting immune function of tolerant microglia in vitro. **a** Representative fluorescent images and **b**, **c** analysis of phagocytic activity determined by fluorescent beads uptake assay in PFF-tolerant microglia with capsaicin treatment (*n* = 3, biological replicates). Scale bar, 25 μm. **d** Representative double-immunofluorescent images for p-α-syn (Ser129) (red) and MAP2 (green) in primary cortical neurons. **e** Quantification of percentage of p-α-syn (Ser129) positive neurons and **f** relative intensity of p-α-syn (Ser129) in primary cortical neurons, which were exposed to conditioned medium from microglia treated with α-syn PFF for 5 days. (*n* = 3, biological replicates). Scale bar, 25 μm. One-way ANOVA with Tukey’s multiple comparisons test was used for statistical analysis. Error bars represent mean ± SD. *p < 0.05, **p < 0.01, ***p < 0.001, ****p < 0.0001

Because capsaicin restored energy metabolism of PFF-tolerant microglia in vitro (Fig. 2), we further hypothesized that a metabolic boost by capsaicin might rescue the impaired immune function of microglia. Phagocytic capacity was significantly reduced in PFF-tolerant microglia but treatment with 10 μM capsaicin significantly increased their phagocytic capacity (Fig. 3a–c). We then tested whether the capsaicin-induced increase in phagocytic capacity of tolerant microglia might reduce the level of phosphorylated α-synuclein in MAP2-positive primary cortical neurons. The levels of p-α-syn (Ser129) induced by PFF in primary cortical neurons were significantly increased after treatment with MCM from PFF-tolerant cells, compared with MCM from microglia acutely stimulated with PFF. MCM from PFF-tolerant cells pretreated with capsaicin significantly reduced the levels of p-α-syn (Ser129) in primary cortical neurons (Fig. 3d–f). These data supported the notion that metabolic boosting by capsaicin might restore phagocytic activity of PFF-tolerant microglia and thus reduce the propagation and aggregation of phosphorylated α-synuclein in neurons.

### Microglia-specific TRPV1 deficiency accelerates PFF-induced PD-like pathology in TRPV1^*flox/flox*^; Cx3cr1^Cre^ mice

**Fig. 4:**
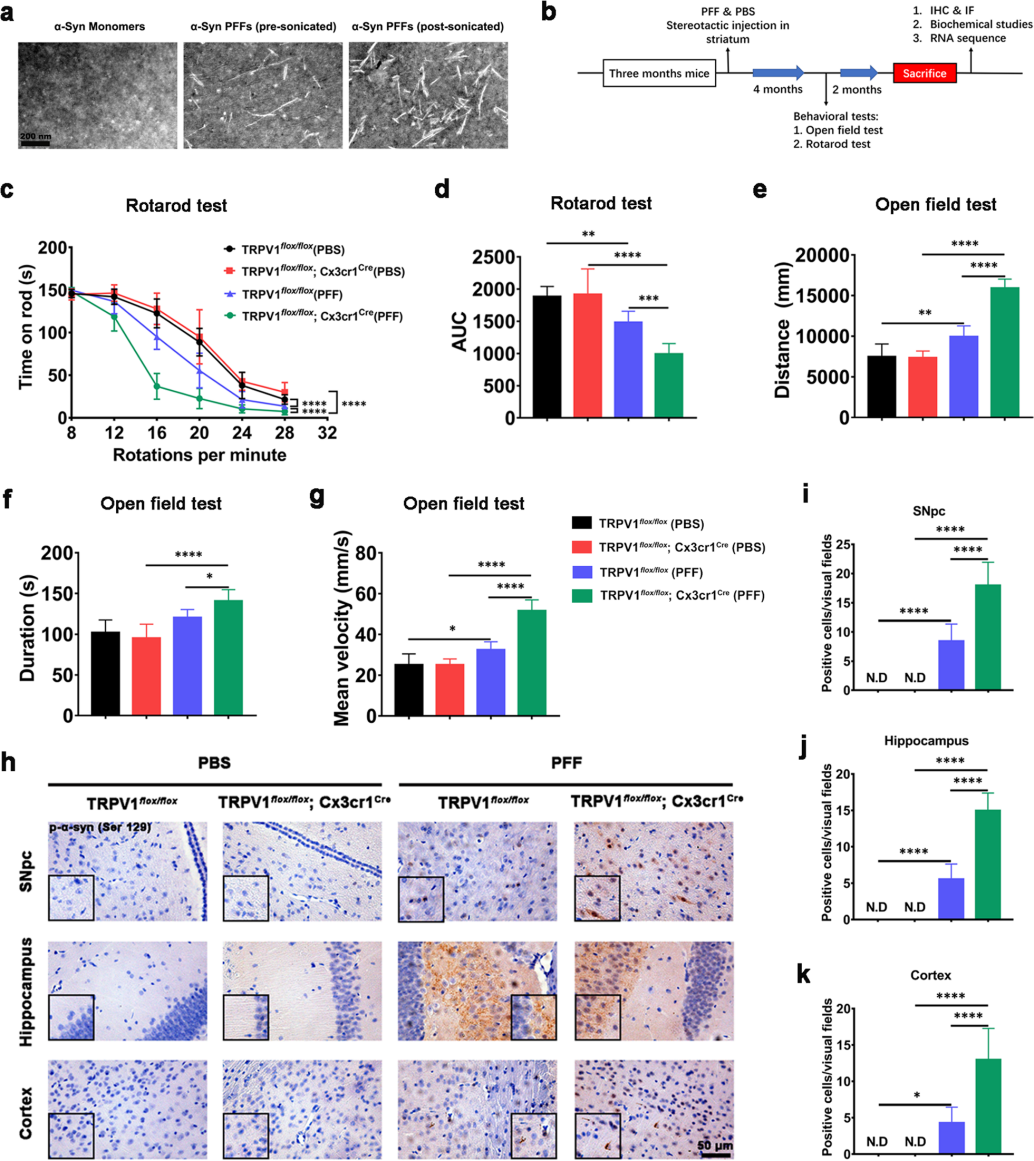
Microglia-specific TRPV1 deficiency accelerates PFF-induced PD-like pathology in TRPV1^*flox/flox*^; Cx3cr1^Cre^ mice. **a** Representative TEM images of α-synuclein monomers, pre-sonicated PFFs and post-sonicated PFFs. Scale bar, 200 nm. **b** Schematic diagram showing PFF experimental design. IF, immunofluorescence; IHC, immunohistochemistry. **c**–**g** Behavioral assessment of TRPV1^*flox/flox*^ and TRPV1^*flox/flox*^; Cx3cr1^Cre^ mice following a single unilateral inoculation of PBS or PFF into the striatum. PFF-injected TRPV1^*flox/flox*^ (*n* = 11) and TRPV1^*flox/flox*^; Cx3cr1^Cre^ mice (*n* = 9), as well as age-matched PBS-injected TRPV1^*flox/flox*^ (*n* = 10) and TRPV1^*flox/flox*^; Cx3cr1^Cre^ animals (*n* = 7) are also shown. **c**–**d** Rotarod test, and **e**–**g** open field test. **h**–**k** Representative immunohistochemistry images **h** and quantification of p-α-syn (Ser129) aggregates in SNpc (**i**), hippocampus **j** and cortex **k** of mice killed 6 months after striatal PFF injection (*n* = 3 animals per group). Scale bar, 50 μm. One-way ANOVA was used for statistical analysis followed by Tukey’s multiple comparisons test in **d**–**k**. Two-way ANOVA was used for statistical analysis followed by Tukey’s multiple comparisons test in **c**. Error bars represent mean ± SD. *p < 0.05, **p < 0.01, ***p < 0.001, ****p < 0.0001

The PFF model of sporadic PD was used to determine the potential neuroprotective function of microglial TRPV1 in PD [29, 30]. PFFs were prepared and verified by TEM [30] (Fig. 4a). Behavioral tests were carried out on TRPV1^*flox/flox*^; Cx3cr1^cre^ mice 4 months after intrastriatal injection of PFF because pathological manifestation in this model became obvious at this time point [24]. Biochemical and behavioral assays, including the rotarod test and open field test, were conducted in TRPV1^*flox/flox*^; Cx3cr1^Cre^ and TRPV1^*flox/flox*^ control mice 6 months after intrastriatal injection of PFF (Fig. 4b).

The rotarod test and open field test were used to monitor the motor coordination of unilaterally PFF-injected mice [18]. Behavioral impairments were accelerated in TRPV1^*flox/flox*^; Cx3cr1^Cre^ + PFF mice compared with TRPV1^*flox/flox*^ + PFF mice (Fig. 4c–g). Compared with TRPV1^*flox/flox*^ + PBS mice, TRPV1^*flox/flox*^ + PFF mice exhibited a decreased retention time on the rod, which was even shorter in TRPV1^*flox/flox*^; Cx3cr1^cre^ + PFF mice (Fig. 4c, d). In the open field test, the travel distance (Fig. 4e), duration (Fig. 4f), and mean velocity (Fig. 4g) of TRPV1^*flox/flox*^; Cx3cr1^cre^ + PFF mice were significantly upregulated compared with TRPV1^*flox/flox*^ + PFF mice.

Previous studies suggested that α-synuclein aggregation propagated from the glossopharyngeal and vagal nerves to the cerebral cortex and neocortex in the advanced stages of disease in PD patients [31]. There is also considerable p-α-syn (Ser129) immunoreactivity in the different brain regions of PFF-injected mice, indicative of α-synuclein pathology [30]. The p-α-Syn (Ser129) immunoreactivity was significantly increased in the SNpc (Fig. 4h, i), hippocampus (Fig. 4h, j) and cortex (Fig. 4h, k) of TRPV1^*flox/flox*^ + PFF mice compared with TRPV1^*flox/flox*^ + PBS mice, and was even higher in TRPV1^*flox/flox*^; Cx3cr1^cre^ + PFF mice. These results suggested that microglial deficiency of TRPV1 aggravated PFF-induced behavioral impairments and propagation of α-synuclein pathology.

### Microglia-specific TRPV1 deficiency accelerates PFF-induced loss of dopaminergic neurons in vivo

**Fig. 5:**
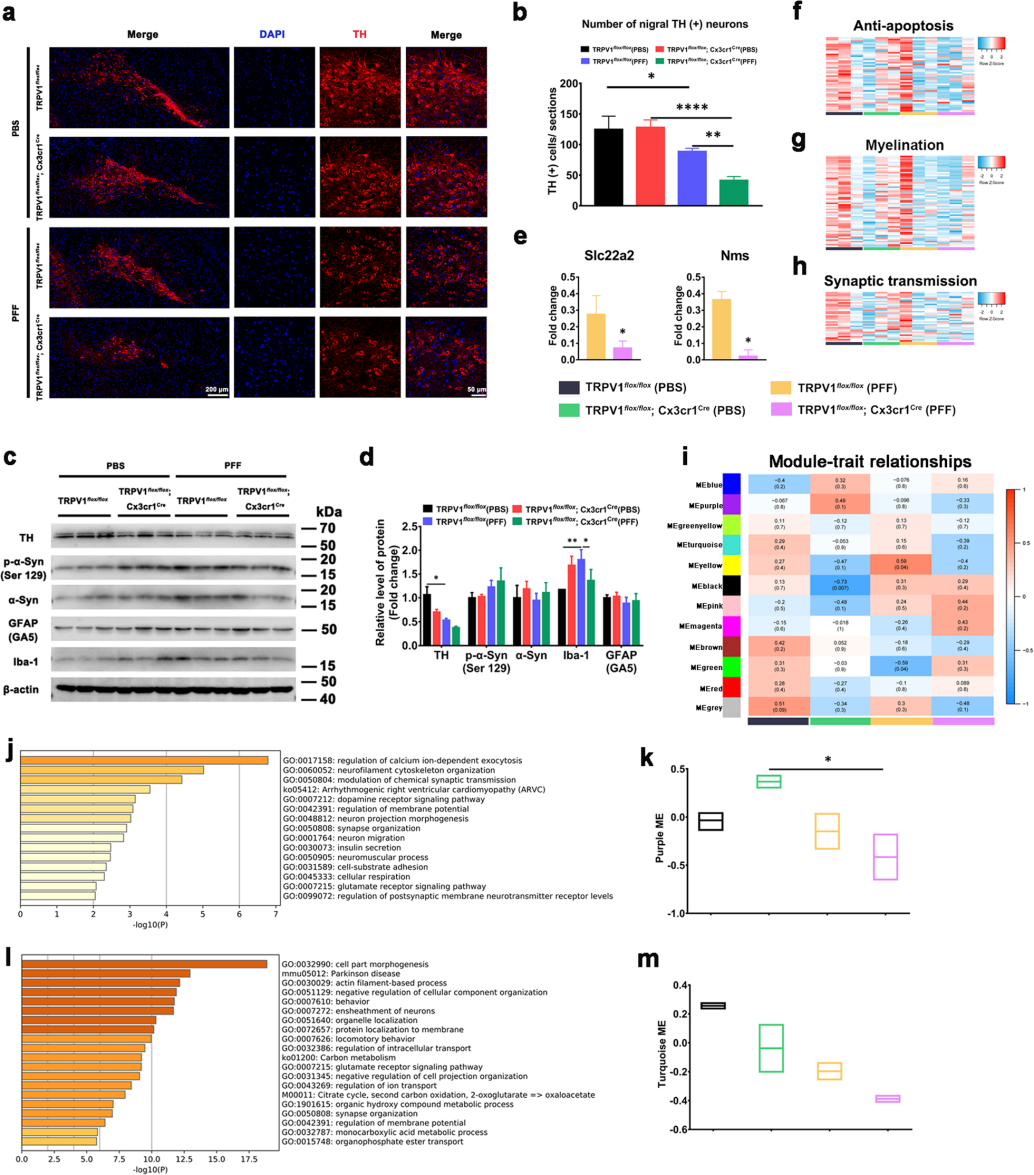
Microglia-specific TRPV1 deficiency accelerates loss of dopaminergic neurons in the SNpc of PFF-injected TRPV1^*flox/flox*^; Cx3cr1^Cre^ mice. **a** Representative images of TH-immunostaining and **b** quantitative analysis of dopaminergic neurons in the SNpc region (*n* = 3, biological replicates). Scale bar, 200 μm or 50 μm, respectively. **c** Immunoblots of TH, p-α-syn (Ser129), α-syn, GFAP (GA5), Iba-1 and β-actin and **d** quantification in SNpc tissues of PBS- or PFF-injected TRPV1^*flox/flox*^ mice or TRPV1^*flox/flox*^; Cx3cr1^cre^ mice (*n* = 3, biological replicates). **e** Differentially expressed genes associated with transmission across chemical synapses (*Slc22a2* and *Nms*) in the brains of TRPV1^*flox/flox*^ or TRPV1^*flox/flox*^; Cx3cr1^cre^ mice 6 months after intrastriatal injection with PFF (*n* = 3, biological replicates). **f**–**h** Heat map representing relative expression levels of genes associated with anti-apoptosis (**f**), myelination **g** and synaptic transmission **h** (*n* = 3, biological replicates). **i** Module–trait relationships between gene modules implicated by WGCNA (*n* = 3, biological replicates). Data are presented as the correlation coefficient (*p* value). **j**–**m** Bar graphs showing the results for GO terms and KEGG pathway enrichment analysis. The top 15 BPs in the purple **j** and top 20 BPs in the turquoise **l** modules are listed. Box plot graphs show the module eigengenes of the purple **k** and turquoise **m** modules. One-way ANOVA with Tukey’s multiple comparisons test was used for statistical analysis in **b**, **d**, k, and **m**. Student’s *t*-test was used for statistical analysis in **e**. Error bars represent mean ± SD. *p < 0.05, **p < 0.01, ****p < 0.0001

Unilateral intrastriatal injections of human PFF resulted in nigrostriatal degeneration of dopaminergic neurons in the SNpc [32]. Consistent with this report, PFF injection resulted in a marked loss of TH positive (TH^+^) neurons in the SNpc of TRPV1^*flox/flox*^ mice, which was aggravated in PFF-injected TRPV1^*flox/flox*^; Cx3cr1^cre^ mice (Fig. 5a, b). Protein expression levels of TH^+^ dopaminergic neurons, p-α-synuclein, α-synuclein, GFAP^+^ astrocytes and Iba-1^+^ microglia in the SNpc of TRPV1^*flox/flox*^ and TRPV1^*flox/flox*^; Cx3cr1^Cre^ mice were measured 6 months after intrastriatal injection of PFF (Fig. 5c, d). As shown in Fig. 5c, the level of p-α-syn (Ser129) was increased, whil numbers of TH^+^ dopaminergic neuron and Iba-1-activated microglia were reduced in the SNpc of TRPV1^*flox/flox*^; Cx3cr1^Cre^ + PFF mice, compared with TRPV1^*flox/flox*^ + PFF mice. A bar graph produced using the RNA sequencing (RNA-seq) data showed that the average fold changes of solute carrier family 22 member 2 (*Slc22a2*) and neuromedin S (*NM’s*) were significantly reduced in PFF-injected TRPV1^*flox/flox*^; Cx3cr1^cre^ mice compared with PFF-injected TRPV1^*flox/flox*^ mice (Fig. 5e). The protein encoded by *Slc22a2* gene is a polyspecific organic cation transporter, which is mainly involved in transmission across chemical synapses. The protein encoded by *Nms* is a member of the neuromedin family of neuropeptides. Expression of genes associated with anti-apoptosis, myelination, and synaptic transmission were markedly downregulated in TRPV1^*flox/flox*^; Cx3cr1^cre^ + PFF mice compared with TRPV1^*flox/flox*^ + PFF mice (Fig. 5f–h).

To further investigate the relationships between microglial TRPV1 and PD pathology, we carried out a WGCNA (Fig. 5i). We identified two gene co-expression modules, the purple (Fig. 5j, k) and turquoise (Fig. 5l, m) modules. Enrichment analysis demonstrated that the deterioration was associated with “dopamine receptor signaling pathway”, “synapse organization” and “behavior” of the TRPV1^*flox/flox*^; Cx3cr1^cre^ + PFF mice compared with TRPV1^*flox/flox*^ + PFF mice, and that the biological processes (BPs) were also associated with “cellular respiration” and “carbon metabolism”. These results, taken together, indicated that microglia-specific deletion of TRPV1 increased the degenerative effects of injected PFF.

### Microglia-specific TRPV1 deficiency exacerbates α-synuclein PFF-induced metabolic defects and immune impairments in vivo

**Fig. 6:**
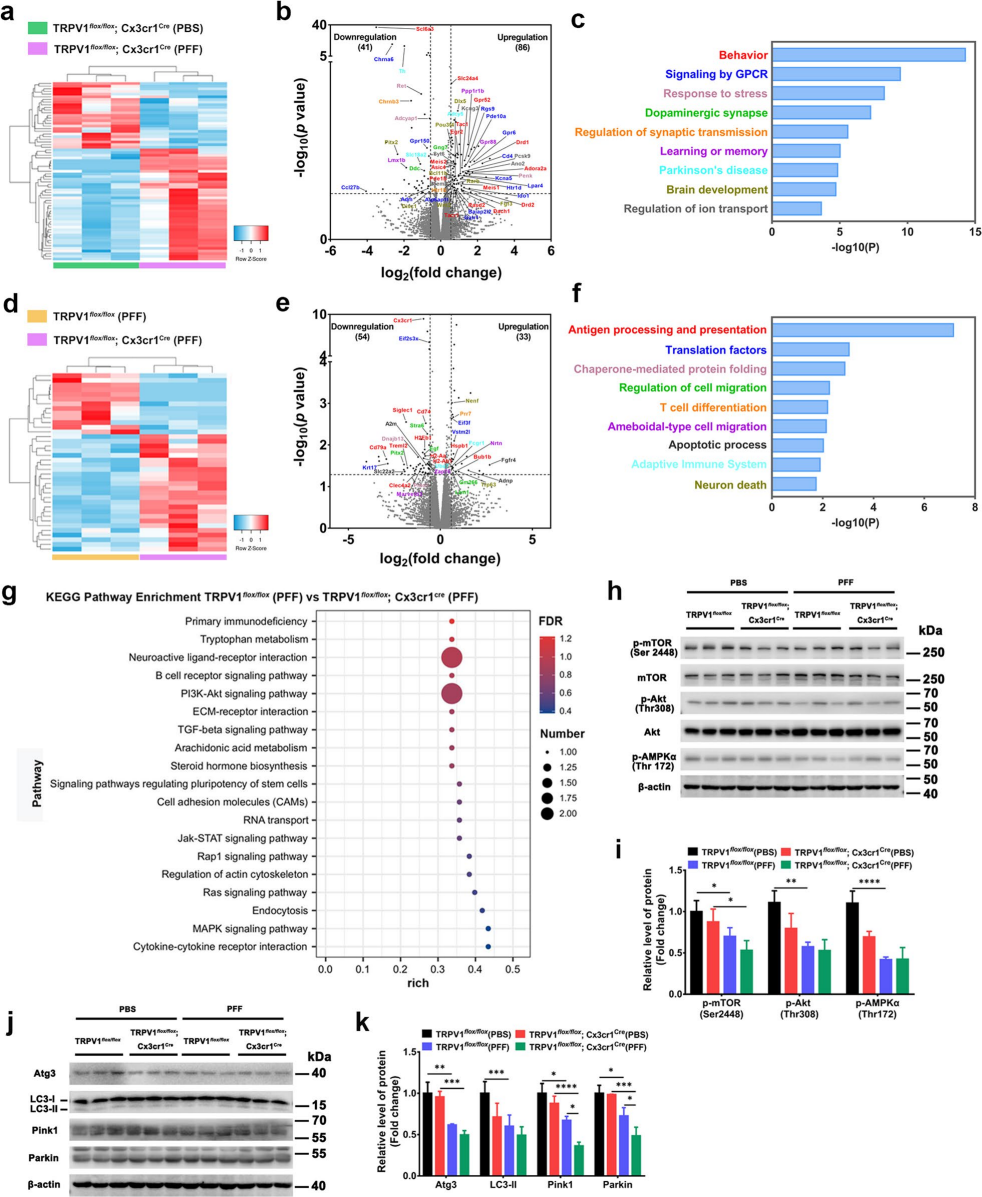
Microglia-specific TRPV1 deficiency accelerates α-syn PFF-induced metabolic defects and immune dysfunction in SNpc of PFF-injected TRPV1^*flox/flox*^; Cx3cr1^Cre^ mice. **a** Two-way heat map cluster analysis, **b** volcano plot (log_2_ (fold change) < − 0.585 or log_2_ (fold change) > 0.585, −log_10_ (*p* value) > 1.30), and **c** GO pathway analysis of differentially expressed genes from PFF-injected TRPV1^*flox/flox*^; Cx3cr1^cre^ mice compared with PBS-injected TRPV1^*flox/flox*^; Cx3cr1^cre^ mice (n = 3, biological replicates). **d** Two-way heatmap cluster analysis, **e** volcano plot (log_2_ (fold change) < − 0.585 or log_2_ (fold change) > 0.585, −log_10_ (*p* value) > 1.30) and **f** GO pathway analysis of differentially expressed genes from PFF-injected TRPV1^*flox/flox*^; Cx3cr1^cre^ mice compared with PFF-injected TRPV1^*flox/flox*^ mice (*n* = 3, biological replicates). **g** Bubble plot of KEGG pathway enriched by differentially expressed genes in brains of PFF-injected TRPV1^*flox/flox*^; Cx3cr1^cre^ mice and TRPV1^*flox/flox*^ mice. **h**–**k** Western blot analysis of metabolic energy pathway (**h**, **i**), and mitophagy markers **j**, **k** in SNpc tissues (n = 3 mice, biological replicates). One-way ANOVA was used for statistical analysis followed by Tukey’s multiple comparisons test. Error bar represent mean ± SD. *p < 0.05, **p < 0.01, ***p < 0.001, ****p < 0.0001

TRPV1 has been implicated in modulation of neuronal function [33, 34], motor behavior [12, 35, 36], immunological function [37] and energy metabolism [11]. To explore the potential effects of microglial TRPV1 during the PD process, we conducted genome-wide RNA-seq analysis. Changes in gene expression between PFF-injected TRPV1^*flox/flox*^; Cx3cr1^cre^ mice and PBS-injected TRPV1^*flox/flox*^; Cx3cr1^cre^ mice are presented as hierarchical clustering (Fig. 6a) and a volcano plot (Fig. 6b). There were 86 upregulated genes and 41 downregulated genes; the enriched BPs were related to behavior, dopaminergic synapse, PD, and learning and memory (Fig. 6c). We next assessed changes in gene expression between PFF-injected TRPV1^*flox/flox*^; Cx3cr1^cre^ mice and PFF-injected TRPV1^*flox/flox*^ mice (Fig. 6d). We found 33 upregulated genes and 54 downregulated genes (Fig. 6e) and 9 significantly enriched BPs, including antigen processing and presentation, translation factors, chaperone-mediated protein folding, regulation of cell migration, T cell differentiation, ameboid-type cell migration, apoptotic process, adaptive immune system and neuronal death (Fig. 6f). KEGG pathway enrichment, including “PI3K–Akt signaling pathway” involved in energy metabolism, “neuroactive ligand–receptor interaction”, “primary immunodeficiency”, “cell adhesion molecules” and “endocytosis” was performed to identify obviously changed pathways between PFF-injected TRPV1^*flox/flox*^; Cx3cr1^cre^ mice and PFF-injected TRPV1^*flox/flox*^ mice (Fig. 6g).

Six months after intrastriatal injection of α-synuclein PFF, protein levels of the mTOR/Akt energy metabolic pathway (Fig. 6h, i) and the mitophagy-associated markers (Fig. 6j, k) in the SNpc of TRPV1^*flox/flox*^ and TRPV1^*flox/flox*^; Cx3cr1^Cre^ mice were measured. There was significant downregulation of expression levels of proteins in the Akt–mTOR–AMPK signaling pathway (Fig. 6h) and mitophagy markers Atg3, LC3, Pink1 and Parkin (Fig. 6j) in the SNpc of TRPV1^*flox/flox*^; Cx3cr1^Cre^ + PFF mice compared with TRPV1^*flox/flox*^ + PFF mice. Together, these results suggested that microglia-specific TRPV1 deletion accelerated metabolic defects and immune impairments that might be responsible for the PFF-induced augmentation of the death of dopaminergic neurons.

### Microglia-specific TRPV1 deficiency exacerbates defects in microglial phagocytosis and immune impairment in SNpc of PFF-injected TRPV1^*flox/flox*^; Cx3cr1^Cre^ mice

**Fig. 7:**
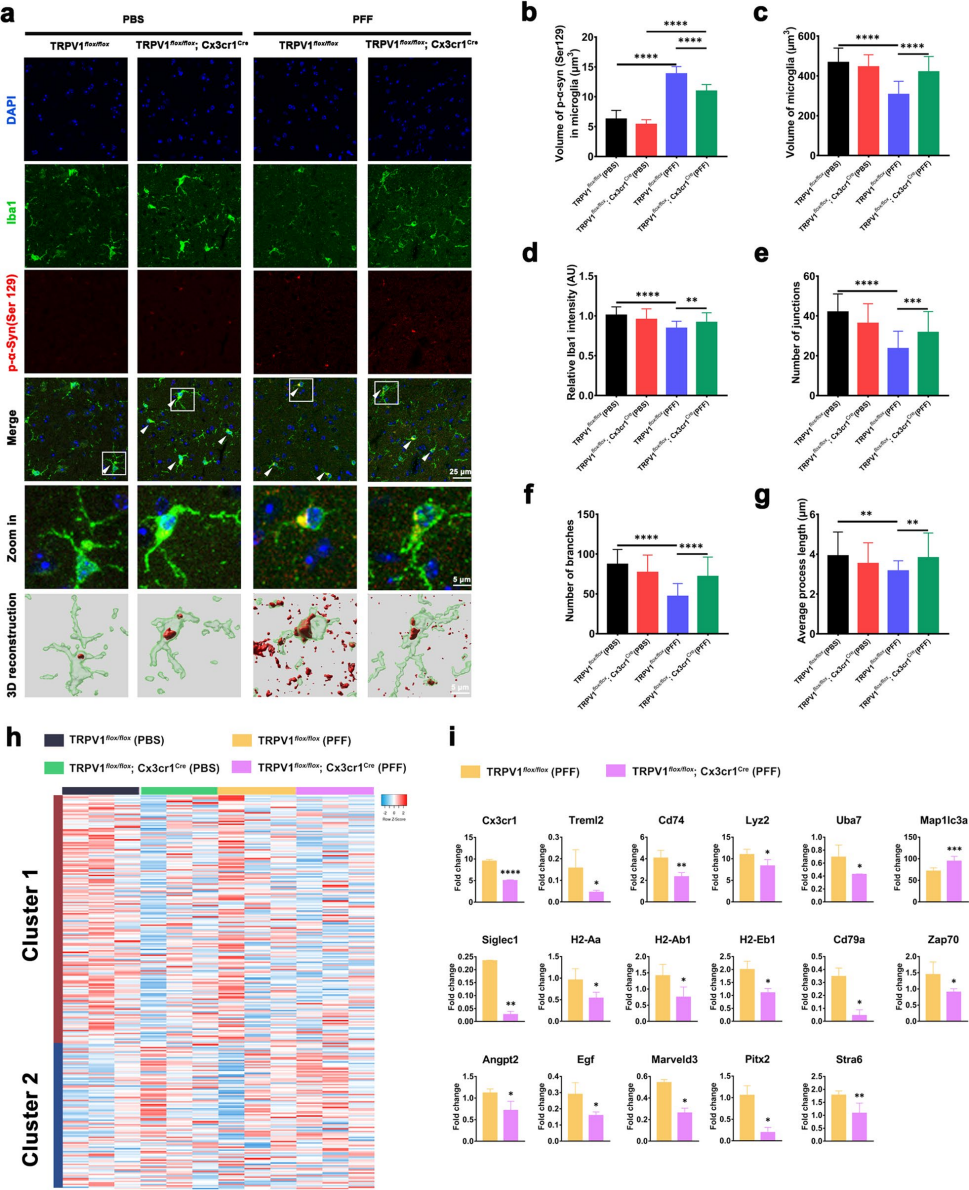
Microglia-specific TRPV1 deficiency attenuates microglial phagocytosis of α-synuclein in the SNpc of PFF-injected TRPV1^*flox/flox*^; Cx3cr1^Cre^ mice. **a** Representative double-immunostaining images for p-α-syn (Ser129) (red) and microglia (green) in the SNpc. The white arrows point to p-α-syn-phagocytosing microglia. Three-dimensional reconstruction of microglia and p-α-syn (Ser129) was produced using Imaris software (*n* = 3, biological replicates). Scale bar, 25 μm or 5 μm, respectively. **b**–**g** Quantification of volume of p-α-synuclein in microglia (**b**), volume of microglia (**c**), relative Iba-1 intensity (**d**), number of junctions (**e**), number of branches (**f**), average length of processes **g** in the SNpc of mice after intrastriatal PFF- or PBS-injection (*n* = 3–4 animals per group). Scale bars, 25 µm and 5 µm for low- and high-magnification images, respectively. **h** RNA-seq analysis of microglial gene expression in PBS- or PFF-injected TRPV1^*flox/flox*^ mice and TRPV1^*flox/flox*^; Cx3cr1^Cre^ mice. Heat map generated by hierarchical gene clustering based on genotypes (vertical, 278 microglial genes; horizontal, individual mouse samples; *n* = 3 mice per group). Cluster 1: proinflammatory genes. Cluster 2: homeostatic genes. **i** Differentially expressed genes associated with immune responses, antigen processing and presentation, and cell motility in the brain of TRPV1^*flox/flox*^ or TRPV1^*flox/flox*^; Cx3cr1^cre^ mice 6 months after intrastriatal injection with PFF (*n* = 3 mice per group). One-way ANOVA with Tukey’s multiple comparisons test **b**–**g**) or Student’s *t*-test **i** were used for statistical analyses. Error bars represent mean ± SD. *p < 0.05, **p < 0.01, ***p < 0.001, ****p < 0.0001

Extensive efforts have elucidated a key role for microglia in ingesting and degrading neuron-released α-synuclein [29, 38, 39]. To determine whether TRPV1 mediated the effects of microglia in PD pathology, we next investigated the interaction between microglia and α-synuclein. Brain sections from TRPV1^*flox/flox*^ control mice and TRPV1^*flox/flox*^; Cx3cr1^Cre^ mice injected with PBS or PFF were stained using Iba-1 antibody and human p-α-syn (Ser129) antibody and a three-dimensional reconstruction of microglia and p-α-synuclein was produced. Immunofluorescent staining showed that p-α-syn (Ser129) protein was more clearly present in Iba-1^+^ microglia at the striatum of TRPV1^*flox/flox*^ + PFF mice, compared with TRPV1^*flox/flox*^; Cx3cr1^Cre^ + PFF mice (Fig. 7a). The volume of p-α-synuclein in microglia was larger in TRPV1^*flox/flox*^ + PFF mice than in TRPV1^*flox/flox*^; Cx3cr1^Cre^ + PFF mice (Fig. 7b). Resting microglia exhibit a ramified morphology, which is transformed into more hypertrophied cell bodies with short processes upon activation [40, 41]. Microglia from TRPV1^*flox/flox*^ + PFF mice exhibited an active phenotype, characterized by a rounded morphology (Fig. 7c, d), with fewer junctions, branches and processes than microglia from TRPV1^*flox/flox*^; Cx3cr1^cre^ + PFF mice (Fig. 7e–g).

An earlier study showed that the activated state of microglia was characterized by upregulation of proinflammatory genes (cluster 1), with concomitant downregulation of normal cell function genes (cluster 2) [42]. Using RNA-seq analysis, we observed a remarkable downregulation of proinflammatory genes (cluster 1) and a concomitant upregulation of genes involved in homeostasis (cluster 2) in the brain of TRPV1^*flox/flox*^; Cx3cr1^cre^ + PFF mice compared with TRPV1^*flox/flox*^ + PFF mice (Fig. 7h). Microglia-specific TRPV1 deficiency resulted in more repressed expression of genes associated with immune responses (Cx3cr1, Treml2, Cd74, Lyz2, Uba7 and Map1lc3a), antigen processing and presentation (H2-Aa, H2-Ab1, H2-Eb1, Cd79a, Zap70 and Siglec1) and cell motility (Angpt2, Marveld3, Egf, Pitx2 and Stra6) in TRPV1^*flox/flox*^; Cx3cr1^Cre^ + PFF mice compared with TRPV1^*flox/flox*^ + PFF mice (Fig. 7i). The protein encoded by *Map1lc3a* is a microtubule-associated protein, which mediates physical interactions between microtubules and components of the cytoskeleton. Upregulated expression of Map1lc3a mRNA thus suggested a blocked autophagic flux and inefficient completion of autophagy. These data supported the conclusion that TRPV1 deficiency accelerated impairment of microglial phagocytic function and hastened immune dysfunction.

## Discussion

Microglia-specific TRPV1 deficiency accelerated PFF-induced behavioral impairment and loss of dopaminergic neurons in vivo*.* TRPV1 deficiency in microglia exacerbated defects in microglial phagocytosis and impairment of immune function in the SNpc of PFF-injected TRPV1^*flox/flox*^; Cx3cr1^Cre^ mice. Using metabolic profiling, we also found that acute stimulation with PFF led to an active microglial phenotype and a switch in cellular metabolism from oxidative phosphorylation to glycolysis via the mTOR–AKT–HIF-1α pathway. Chronic exposure of microglia to PFF induced innate immune tolerance and metabolic defects, including changes in oxidative phosphorylation and aerobic glycolysis. Capsaicin rescued impaired cellular metabolism, mTOR signaling and immune functions of PFF-tolerant microglia. These results indicated that TRPV1 was an important therapeutic target for restoring microglial metabolic immune function for the treatment of PD.

The brains of PD patients contain intracellular aggregated α-synuclein, a 140-amino-acid cytoplasmic protein that is located in presynaptic nerve terminals and participates in the assembly of SNARE complexes [43]. In PD, α-synuclein phosphorylated at residue Ser 129 polymerizes to form protein aggregates that deposit within the brain. Because mutations of the gene encoding α-synuclein cause early-onset PD, α-synuclein is believed to be involved in the pathogenic processes of synucleinopathy. Mounting evidence has shown that α-synuclein can be transmitted between neurons by a progressive “prion-like” mechanism, leading to protein aggregates within the brain [44].

Microglia, the brain’s innate immune cells, play a key role in ingesting and degrading α-synuclein released by neurons [29, 38, 39]. The efficiency of the microglial autophagy–lysosome degradation system is decreased in PD, promoting accumulation of misfolded α-synuclein and causing degeneration of dopaminergic neurons [10]. p-α-Syn (Ser129) protein was clearly present in microglia at the striatum of TRPV1^*flox/flox*^ + PFF mice and ﻿the volume of microglial p-α-synuclein was larger in TRPV1^*flox/flox*^ + PFF mice than in TRPV1^*flox/flox*^; Cx3cr1^Cre^ + PFF mice (Fig. 7a, b). These data showed that microglia-specific TRPV1 deficiency exacerbated defects in microglial phagocytosis and immune impairment in vivo.

﻿Pathological aggregates of α‐synuclein disrupt synaptic protein trafficking, as well causing neuroinflammation and deterioration in the functions of autophagy–lysosomes and mitochondria [45–47]. Aggregated α‐synuclein can be released from a donor cell to a neighboring neuron or glial cell; both intracellular and intercellular toxicity of aggregated α‐synuclein then accelerate cellular damage [48–53]. Regulation of lipid rafts, endocytosis, trans-synaptic transmission and direct penetration by Parkin have been shown to mediate the spread of aggregated α‐synuclein [45, 54]. Autophagic dysfunction has also been suggested to accelerate intercellular transfer of α‐synuclein [55–57]. Expression of both the mitochondrial protein kinase Pink1 and the cytoplasmic ubiquitin ligase Parkin was downregulated in PFF-tolerant microglia compared with acutely stimulated microglia. Activation of TBK1 by phosphorylation on its activation loop site (Ser172), a feed-forward amplification mechanism to promote mitochondrial clearance, was downregulated in PFF-tolerant microglia. Chronic treatment with PFF reduced TRPV1 expression in microglia (Fig. 2f) and treatment of PFF-tolerant microglia with capsaicin increased the expression of Pink1, Parkin, p-TBK1/NAK (Ser172) and TRPV1 (Fig. 2g).

It was reported that AMPK involved in oxidative stress process as a redox-sensitive protein [58, 59]. As shown in Fig. 2d, ROS production was significantly upregulated in both acute and chronic PFF-stimulated microglia (Fig. 2d). Previous studies have demonstrated that AMPK was sensitive to cellular stress and phosphorylation of AMPKα at Thr172 was decreased in a ROS-dependent manner during aging and obesity [60, 61]. This might explain that phosphorylation of AMPKα at Thr172 was significantly downregulated in chronic PFF-induced tolerance (Additional file 1: Fig. S1a, b).

Metabolic dysfunction and redox stress have been shown to be the key mediators of proteotoxicity associated with PD. Mitochondria are important targets of α‐synuclein, and mitochondrial function was perturbed in both cell culture and transgenic mouse models of PD [62–64]. After exposure to the pesticides Maneb and paraquat, aggregated α‐synuclein induced release of cytochrome c from mitochondria to the cytosol [65]. When microglial gene expression profiles were assessed in the brains of TRPV1^*flox/flox*^; Cx3cr1^cre^ + PFF mice and TRPV1^*flox/flox*^ + PFF mice using RNA-seq analysis, we observed that microglia-specific TRPV1 deficiency resulted in greater repression of genes associated with immune responses, antigen processing and presentation, and cell motility in TRPV1^*flox/flox*^; Cx3cr1^Cre^ + PFF mice compared with TRPV1^*flox/flox*^ + PFF mice (Fig. 7i).

Loss of key mitophagy proteins that are involved in mitochondrial clearance has been shown to cause PD, as well as a significant proportion of genes encoding PD proteins associated with the autophagy–lysosomal pathway. Enhancement of mitophagy has also been reported to abolish the neuronal hyperphosphorylation of tau in Alzheimer’s disease and rescue memory impairment in transgenic mice.

## Conclusions

In conclusion, our findings suggested that defects in microglial phagocytosis and impaired immune function were pivotal events in the pathogenesis of PD and that microglial metabolic immune functions represented a potential target for therapeutic intervention.

## Supplementary Information

**Additional file 1: Fig. S1.** p-AMPKα (Thr172) expression in microglia after chronic treatment with PFF. **a**, **b** Immunoblot analysis of p-AMPKα (Thr172) in microglia after treatment with control, acute PFF or chronic PFF (*n* = 4 per group). One-way ANOVA with Tukey’s multiple comparisons test was used for statistical analysis. Error bars represent mean ± SD. ****p < 0.0001.

## Data Availability

The authors declare that the data supporting the findings of this study are available from the corresponding author upon reasonable request.

## Abbreviations

PD: Parkinson’s disease
PFF: Preformed fibril
TRPV1: Transient receptor potential vanilloid 1
TEM: Transmission electron microscopy
WGCNA: Weighted gene co-expression network analyses
MCM: Primary microglia-conditioned medium
DAPI: 4′,6-Diamidino-2-phenylindole
DMEM: Dulbecco’s modified Eagle’s medium
GFAP: Glial fibrillary acidic protein
Iba-1: Ionized calcium-binding adapter molecule 1
IL-1: Interleukin 1
LPS: Lipopolysaccharide
MAP2: Microtubule-associated protein 2
ROS: Reactive oxygen species
PBS: Phosphate-buffered saline
SNpc: Substantia nigra pars compacta
TH: Tyrosine hydroxylase
TNF: Tumor necrosis factor
