# Supplementary figures and images for: A breakdown in microglial metabolic reprogramming causes internalization dysfunction of α-synuclein in a mouse model of Parkinson’s disease
Source: J Neuroinflammation. 2022 May 22;19:113. doi: 10.1186/s12974-022-02484-0 (PMC9124408; doi:10.1186/s12974-022-02484-0)

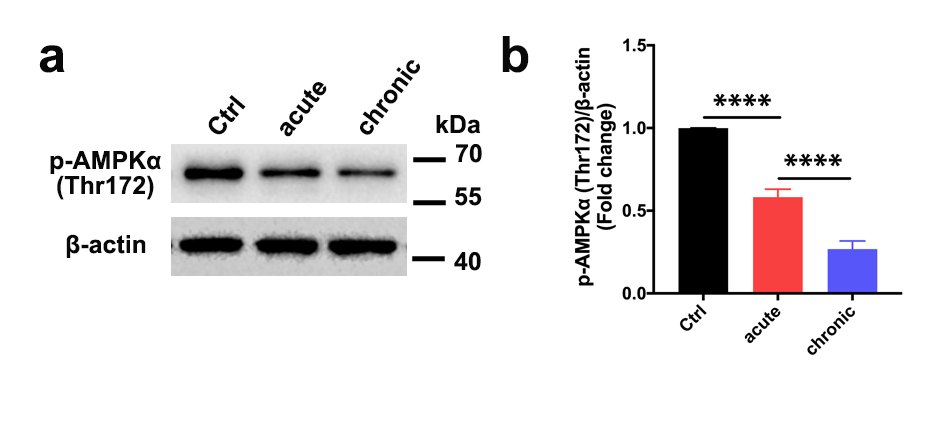

Supplement: Supplementary file 1 — Additional file 1: Fig. S1. p-AMPKα (Thr172) expression in microglia after chronic treatment with PFF. a, b Immunoblot analysis of p-AMPKα (Thr172) in microglia after treatment with control, acute PFF or chronic PFF (n = 4 per group). One-way ANOVA with Tukey’s multiple comparisons test was used for statistical analysis. Error bars represent mean ± SD. ****p < 0.0001. [file 12974_2022_2484_MOESM1_ESM.tif]
